# Home Literacy and Numeracy Interact and Mediate the Relationship Between Socio-Economic Status and Early Linguistic and Numeracy Skills in Preschoolers

**DOI:** 10.3389/fpsyg.2021.662265

**Published:** 2021-09-29

**Authors:** Paola Bonifacci, Diego Compiani, Alexandra Affranti, Benedetta Peri

**Affiliations:** Department of Psychology, University of Bologna, Bologna, Italy

**Keywords:** socio-economic status, early literacy, early numeracy, home literacy, home numeracy

## Abstract

This longitudinal study aimed at evaluating the relationships between socio-economic status (SES) and early literacy and numeracy skills, testing home literacy and home numeracy as mediators. It also investigated the interaction of home literacy and numeracy on early literacy and numeracy skills. The study involved 310 preschool children attending the second and the third year. Parents completed questionnaires on SES and home literacy and numeracy. In the first session, children were administered language measures and non-symbolic numeracy skills and, in the second wave, tasks of early literacy and symbolic numeracy skills. Structural equation models (SEMs) showed that SES was predictive of early language and literacy skills and non-symbolic numeracy skills. In addition, home literacy and home numeracy significantly mediated the relationships between SES and children’s skills. Finally, home literacy and home numeracy showed a significant negative interaction on symbolic numeracy skills. Implications for research and educational settings are discussed.

## Introduction

The role of the home environment in early literacy and numeracy development has received progressively increasing attention, also according to theoretical models such as Neuroconstructivism (Westermann et al., 2007), that emphasize the role of environmental variables on children’s cognitive development. Children who grow up in families with low socio-economic status (SES) often exhibit delays in school readiness. These delays might undermine their academic goals and might lead to a lifelong trajectory of underachievement, school dropout, and underemployment, compared to children from families of higher SES (Bradley and Corwyn, 2002). Delays in school readiness are part of a larger set of disparities associated with low SES, which confers elevated risks in diverse areas of behavioral skills (Adler and Newman, 2002) and health outcomes (Case et al., 2002). Sirin (2005) defines SES as “*the individual’s or a family’s ranking on a hierarchy according to access to or control over some combination of valued commodities such as wealth, power, and social status*” (Sirin, 2005, p. 418). Bollen et al. (2001) suggest that (SES) “*refers to the position of individuals, families, households, or other aggregates on one or more dimensions of stratification. These dimensions include income, education, prestige, wealth, or other aspects of standing that members of society deem salient.”* (p. 157). However, the debate on how to operationalize SES is still ongoing. The choice of SES measure usually includes education, occupation, and income (Brese and Mirazchiyski, 2013), but also possessions (e.g., books and personal computers) and, in some cases, the concept extends to cultural and social capital (e.g., relationships). The choice of measures might depend on many factors (Broer et al., 2019), such as the conceptual relevance at the time of the study, the applicability of the measure to the specific populations being studied, and comparability with measures used in other studies. Parental education has been identified as the index with the strongest associations with children’s educational outcomes (Davis-Kean, 2005), strongly correlated with other important SES indicators (Sirin, 2005). As suggested by Hannon et al. (2020), disadvantage does not inhere in individuals; but might be the results of relationships between individuals, society, and the school system. It is, therefore, possible that disadvantaged families’ literacy does not match school literacy as closely as does advantaged families’ literacy. Living in poverty predicts parents’ resource strain in terms of both material resources and emotional and psychological resources, possibly impacting the quantity and quality of home literacy and numeracy activities the children are exposed to Elliott (2020). In this regard, many studies have found evidence of a positive relationship between home literacy and early literacy skills (Sénéchal et al., 1998) and between home numeracy and early numeracy skills (Skwarchuk et al., 2014). However, past research demonstrates that associations between SES and home literacy are typically moderate in magnitude (Linver et al., 2002; Davis-Kean, 2005; Mistry et al., 2010; Elliott, 2020), suggesting high levels of variability within both low- and high-SES families in their support for home learning.

Furthermore, less evidence has been collected about the relationship between SES, home numeracy, and early numeracy skills. Finally, although many studies investigated the effects of SES on school achievements longitudinally (see for a review Bradley and Corwyn, 2002), fewer longitudinal studies are available that consider the impact of SES on specific literacy and numeracy subdomains in preschool years, including the home learning environment as mediator (e.g., Park, 2008; Elliott, 2020). Further, no evidence is available in this regard for the Italian context. Based on these considerations, in a sample of Italian preschoolers, the present study investigated how SES is related to different subdomains of language and literacy skills and symbolic and non-symbolic numeracy skills in preschool children in two different time moments, the second and third year. Further, it analyzed the interaction of home literacy and numeracy on early literacy and numeracy skills.

### SES, Early Literacy and Numeracy Skills, and Home-Related Activities

Considering the influence of SES on development, children from low SES families have been found to lag behind their high-SES peers in language skills, such as vocabulary, grammar, narrative skills, phonological awareness, speed of language processing (see Hoff, 2013 for a review), listening comprehension (Bonifacci et al., 2020), and reading attitudes (Hemmerechts et al., 2017). There is also some evidence that SES might be related to math skills (e.g., Reardon and Portilla, 2016), although less evidence has been collected in this regard and with somehow contrasting results.

Previous studies have shown that parental involvement at home is unequally distributed by SES (Bradley and Corwyn, 2002; Buckingham et al., 2014). Hemmerechts et al. (2017) also found that children with a lower SES experience more late parental involvement in literacy activities than children with a higher SES and that late parental involvement in literacy activities is an adjustment for worse or better reading literacy during primary school. Thus, SES levels might influence cognitive and literacy development mediating the educational opportunities that can be achieved (e.g., exposure to books, reading practice, quality of schools, etc.), with possible long-term outcomes (Suggate et al., 2018). It has been suggested that home activities may serve as a buffer that promotes resilience in the context of low-SES (Benzies and Mychasiuk, 2009), although literature reports diverse effects sizes and patterns of moderators (Inoue et al., 2020).

Considering language and literacy skills, Hoff (2003) found a higher degree of lexical diversity (type/token ratio) and more complex utterances (mean length of utterances, MLUs) in children of high-SES mothers, compared to those with lower SES. Further, SES levels might influence linguistic and literacy development mediating the educational opportunities that can be achieved (e.g., exposure to books, reading practice, quality of schools, etc.). The quality of the home literacy environment (Sénéchal et al., 1998) is a good predictor of children’s literacy attainments. van Steensel (2006) followed a sample of children in the Netherlands from the end of kindergarten until second grade and found a tendency toward an enriched home literacy environment as the educational level (EL) of the mother increased. Sénéchal (2006) suggested that home literacy experiences might be viewed as proximal variables that can directly affect child outcomes, whereas SES should be considered a distal cause.

Considering the relationship between SES and math skills, most authors suggested a positive relationship (Duncan and Magnuson, 2011; Reardon and Portilla, 2016), although there are more minor studies than the literacy domain and significant differences between countries and different school systems (Baird, 2012). In addition, SES disparities are differently related to subcomponents of numeracy skills, with higher discrepancies in the verbal and symbolic aspects of numeracy skills and minor differences in performance in non-verbal and non-symbolic tasks (for a review, see Jordan and Levine, 2009). There was also contrasting evidence concerning the relationship between SES and the quantity of home learning activities (Elliott and Bachman, 2018). Silinskas et al. (2010) showed that mothers and fathers with low SES backgrounds reported more teaching of reading and numeracy than mid-SES parents; also, the lower the children’s academic performance at the beginning of primary school, the more teaching by mothers and fathers was reported. These results suggest that parents might adaptively adjust the frequency of home literacy and numeracy activities to the child’s performance level, even when they had low SES. Similar results were found by LeFevre et al. (2010) and Niklas and Schneider (2014). There are, however, studies that did not find a relationship between SES and home numeracy (de Keyser et al., 2020). Others instead found that higher SES children were exposed to higher quality numeracy activities than lower SES children (DeFlorio and Beliakoff, 2015).

Finally, SES might differently interact with home literacy/numeracy effects. For example, Leyva et al. (2017) found that maternal writing support predicted gains in children’s reading skills in ethnically diverse low-income mothers. Still, numeracy support did not predict improvements in children’s math skills.

### Early Literacy and Numeracy Skills

Complex mathematical abilities and mature literacy skills (decoding and reading comprehension) are trained at school, but during preschool years children already spontaneously develop basic calculation skills (Levine et al., 1992) and show literacy-related skills such as phonemic awareness and letter knowledge (Ehri et al., 2001). Also, it has to be underlined that literacy and numeracy skills are related (Cirino et al., 2018; Koponen et al., 2020), and some studies found that early literacy predicts early numeracy skills (Tobia et al., 2016) and later mathematical skills (Hecht et al., 2001).

Early numeracy abilities involve the understanding of magnitudes and the development of numerical processing skills and are manifested during the first few months of life in humans from various cultural backgrounds (Xu et al., 2005). The approximate number system (ANS) (Dehaene, 1992, 2001) is a core mechanism involved in number processing that allows to quickly understand, approximate, and manipulate numerical quantities. Based on von Aster and Shalev’s (2007) model, non-symbolic numerical processing is followed by the acquisition of verbal labels for numbers, and then the child progressively acquires the written code for numbers. Previous literature has documented that the main predictors of math skills from preschool to primary school include quantity comparison (Clarke and Shinn, 2004) and number knowledge (Göbel et al., 2014). In addition, some studies found a specific effect of other basic number skills such as size seriation and counting (Tobia et al., 2016). From a developmental perspective, some studies proposed that language is essential for the growth of numerical competencies (Hauser et al., 2010), and mathematical language was found to be a unique significant predictor of numeracy performance (Purpura and Logan, 2015). The relationship between non-verbal approximate numerical abilities and symbolic number knowledge is, therefore, controversial. Some authors suggest that ANS forms a crucial conceptual foundation for understanding symbolic number words (Gallistel and Gelman, 1992, 2000; Wagner and Johnson, 2011). However, other studies failed to find a relationship between the two (Huntley-Fenner and Cannon, 2000).

As far as literacy is concerned, previous reviews have outlined how letter knowledge and phonemic awareness represent strong predictors of later decoding skills (Torppa et al., 2006; Caravolas et al., 2013; see Bellocchi et al., 2017 for Italian). Conversely, general linguistic skills, such as vocabulary and morpho-syntactic comprehension, might act as first precursors of the emergence of early literacy skills (phonological awareness and letter knowledge) but are instead considered a direct longitudinal predictor of later reading comprehension skills (Foorman et al., 2015; Hulme et al., 2015).

### Home Literacy and Home Numeracy

Many studies have addressed the role of home literacy activities in literacy development (Evans et al., 2000; Sénéchal and LeFevre, 2002; Foy and Mann, 2003; Hood et al., 2008; Stephenson et al., 2008) and that of home numeracy activities in numeracy development (Blevins-Knabe and Musun-Miller, 1996; Pan et al., 2006; LeFevre et al., 2009; Kleemans et al., 2012, 2016). Most of the literature on home literacy and home numeracy was obtained through parents’ self-report questionnaires (e.g., Sénéchal and LeFevre, 2002; LeFevre et al., 2009), suggesting that parents’ reports can be considered suitable tools in this research field (Sim et al., 2019).

Previous research found that home literacy, that is, exposure to books and reading in the familiar context, is positively related to early language skills such as expressive and receptive language (Payne et al., 1994; Roberts et al., 2005) and early literacy skills, such as letter knowledge, phonological awareness (Evans et al., 2000; Foy and Mann, 2003; Hood et al., 2008; Stephenson et al., 2008). Some studies also suggested that parents may also foster the development of writing competence (Wollman-Bonilla, 2001; Reutzel et al., 2005; Saint-Laurent and Giasson, 2005; Puranik et al., 2018; Hofslundsengen et al., 2019; Guo et al., 2020). In addition, the Home Literacy Model (Sénéchal and LeFevre, 2002; Sénéchal, 2006; Sénéchal et al., 2017) suggests that parent–child interactions on code-related activities, such as the teaching of reading and spelling (formal activities), are related to reading development. In contrast, meaning-related activities, such as parents’ shared book reading with their children (informal activities) (Sénéchal, 2006), are predictors of oral language skills and later reading comprehension (Hulme et al., 2015).

However, some contrasting results are reported in the literature on the relationship between home literacy and early literacy skills. A study by Inoue et al. (2020), conducted on children from first to second grade, found that home literacy formal activities were associated with better letter knowledge or phonological awareness in Dutch and Greek, while access to literacy resources was related to emergent literacy skills in all languages. On the counterpart, informal activities such as shared book reading did not predict any cognitive or early literacy skills in any language. Bonifacci et al. (2021) did not find direct relationships between home literacy and early literacy skills in a path model including cognitive skills, although the two domains had significant correlation indexes.

Indeed, many pieces of evidence now indicate that parents also matter in the development of children’s numeracy skills and recognize the influential role of home numeracy activities (LeFevre et al., 2009), defined as the parent–child interactions that include experiences with numerical content in daily-life settings (Mutaf Yildiz et al., 2018). Considering the role of home numeracy in early numeracy skills, positive relationships have been found (Blevins-Knabe and Musun-Miller, 1996; Pan et al., 2006; LeFevre et al., 2009; Kleemans et al., 2012, 2016; Bonifacci et al., 2021). Home numeracy can be conceived as a multifaceted domain, and its relationship with children’s numeracy skills might be differentiated based on direct (formal) versus indirect (informal) activities (LeFevre et al., 2009; Skwarchuk et al., 2014). Direct activities focus on counting and teaching numbers and have been found to be related to children’s symbolic numeracy abilities. In contrast, indirect activities have been found to be related to children’s non-symbolic numeracy abilities. They involve playing games with numbers (e.g., dice) or doing everyday activities where you need to count. Other authors also highlighted the importance of “math talk,” which can be considered another aspect of home numeracy and is referred to how parents use number words in everyday life (Braham et al., 2018). Elliott et al. (2017) found that parents’ use of numbers larger than 10 was positively and significantly related to children’s numeracy skills even when controlling for parents’ overall talk. It has also been found that intervention directed to parents leads to enhanced home numeracy activities and significant gains in children’s early numeracy skills (Niklas and Schneider, 2014). However, some studies did not find a significant association between home numeracy and children’s early numeracy skills (e.g., Blevins-Knabe et al., 2000; de Keyser et al., 2020). Other studies showed differential effects of formal and informal home numeracy activities on different domains of number processing skills (Manolitsis et al., 2013; Kleemans et al., 2016; Mutaf Yildiz et al., 2018). Mutaf Yildiz et al. (2018) found that formal home numeracy was related to enumeration skills; informal home numeracy was related to calculation and symbolic processing, but there were no relationships with non-symbolic processing. In a meta-analysis by Mutaf- Yildiz et al. (2020), it was concluded that only advanced home numeracy interactions were associated with children’s numeracy skills, but not basic ones.

A relatively minor number of studies have directly investigated the cross-domain effects of home literacy on numeracy and those of home numeracy on literacy. These studies tried to understand whether home-learning experiences might have specific effects only on their direct domains (home literacy for literacy and home numeracy for numeracy) or, instead if there are cross-domains effects, that is home literacy affecting early numeracy skills and home numeracy affecting early literacy skills (Melhuish et al., 2008; Skwarchuk et al., 2014). Baker (2014) found that the home literacy environment was related to reading but not numeracy in Mexican preschool children, whereas other studies have reported that numeracy skills are associated with home literacy experiences at least as strongly as with home numeracy experiences (LeFevre et al., 2009, 2010; Anders et al., 2012). Similarly, Soto-Calvo et al. (2020) found that home literacy was predictive of numeracy skills. Huntsinger et al. (2016) demonstrated that home numeracy activities predicted both numeracy and literacy skills, both concurrently and longitudinally, whereas home literacy activities predicted reading scores concurrently. Similarly, Napoli and Purpura (2018) reported that the home literacy environment was not broadly predictive of children’s literacy and numeracy skills, but they found that the home numeracy activities predict a specific aspect of children’s literacy development (vocabulary). Bonifacci et al. (2021) found that home numeracy was directly linked to early numeracy, but in their SEM model, there was no reciprocal interaction between home literacy and numeracy skills and between home numeracy and literacy skills. In this study, however, cognitive skills of executive functions (EFs) and working memory were also included in the model.

### The Present Study

Within a longitudinal design involving 4- and 5-years old children attending preschool, the present study was aimed at evaluating the relationships of SES with early language and literacy skills and that of SES with early non-symbolic and symbolic numeracy skills, considering the role of home literacy and numeracy as potential mediators. Further, we evaluated the interaction of home literacy and numeracy on early language/literacy and symbolic/non-symbolic numeracy skills, including SES as a potential mediator. Home literacy and home numeracy were evaluated as single constructs and included both direct (formal) and indirect (informal) activities. To fulfill the project’s aim, we administered children two different sets of measures in the first and second waves of assessment. In the first wave, we evaluated measures that were thought to be adequate for the age range, and that first emerge in the developmental trajectory of literacy and numeracy skills. Therefore, we included vocabulary and morpho-syntactic comprehension as a proxy of language skills and non-symbolic quantity comparison and seriation as precursors of numeracy skills. Then, in the second wave, we choose measures of letter knowledge, early writing, and phonological awareness as indexes of early literacy skills and symbolic number recognition and biunivocal correspondence as indexes of early numeracy skills.

The main objectives and expected results of the study were the following:

1. Considering SES, we aimed to evaluate if it predicts both home literacy and numeracy activities and children skills. Based on previous studies, strong evidence suggests that SES predicts early language skills (vocabulary, morpho-syntactic comprehension) and, in turn, early literacy skills. However, for the latter, an intervening role of school activities might damper the influence of SES. Concerning early numeracy, minor evidence is available and reported high variability between countries and different school systems (Baird, 2012). Some authors suggested a more substantial role of SES for symbolic, rather than non-symbolic, numeracy skills. Within this framework, we expect SES to predict both early language and literacy skills. We also expect a relationship between SES and early numeracy skills, although possibly lower than the relationship between language and literacy measures. Further, we expect stronger relationships in the first wave of assessment due to the potential intervening role of school activities on the second wave. Finally, we also expect SES to have a direct relationship with both home literacy and home numeracy (Jordan and Levine, 2009).
2. Regarding home literacy and home numeracy activities, we expect direct relationships between home literacy and language and literacy skills and between home numeracy and non-symbolic and symbolic numeracy skills. Further, we aim to evaluate if they mediate the role of SES on children’s skills.
3. Finally, we aim to evaluate the interaction’s effect between home literacy and numeracy on children’s literacy and numeracy skills, including SES as a mediator. Since relatively more studies reported an effect of home literacy on numeracy compared to that of home numeracy on literacy, we expect the interaction to be associated with numeracy skills rather than with language and literacy skills.

Previous results on the Italian context did not find a direct relationship between home literacy and early literacy skills, nor evidence of an interaction between language and numeracy skills. Therefore, variations due to different socio-educational contexts might be expected with respect to previous literature.

## Materials and Methods

### Participants

A total of 310 Italian monolingual children (females = 55.2%) were involved in the study in two times points: in the spring of the second year of preschool (mean age = 56.95 months ± 3.66), and 1 year later, in the spring of the third year of preschool (mean age = 68.55 months ± 3.47). Parents of children received the questionnaires about SES and home literacy and numeracy. All children attended a public all-day preschool program in Italy where the Laboratory for the Assessment of Learning Disabilities (LADA) of Bologna University’s Department of Psychology was running the LOGOS project, funded by the Municipality of Bologna, which is aimed at the early identification of literacy and numeracy skills. None of the children had been referred to neuropsychiatric units for any range of developmental disorders or sensory or neurological impairments. Thus, the sample was relatively homogeneous for educational exposure, considering that all the teachers received training on early literacy and numeracy skills within the project. The Italian preschool program is a 3-year program that involves children from 3 to 6 years. During these preschool years, formal instruction regarding literacy and numeracy skills is not provided. However, children are engaged in activities that are aimed at improving socialization and numeracy and linguistic development.

The parents of all children involved in the study gave their informed consent, and the Bioethical Committee of the University of Bologna approved the LOGOS project (prot. 1470, October 2, 2017).

### Materials

#### Background Information

Information regarding the parents’ socio-EL and occupation was collected and scored, according to the Four Factor Index of Social Status (SES) (Hollingshead, 2011), to achieve a composite score for each child’s SES. For the present study, indexes of EL and occupation (O) were used. Thus, a score from 1 to 7 is given for EL and a score between 1 and 9 for occupation. SES scores for fathers and mothers were then calculated according to the formula EL × 3 + O × 5, and a compound SES score for children derived from the mean of the two values.

#### Home Literacy and Home Numeracy Questionnaire

A questionnaire assessing home literacy and home numeracy activities was administered to parents. Parents could complete it together or by who spends more time with the child, usually the mother. In line with other studies that adopted a similar approach, we opted for a short questionnaire that is easy to fill out by parents to encourage greater adherence to the study (Stephenson et al., 2008; Manolitsis et al., 2013; see also Bernabini et al., 2020b; Bonifacci et al., 2021). The questionnaire included four questions on home literacy activities. Two were referred to more formal activities [“How often do you and your child read or write letters of the alphabet?”; “How often do you and your child use games (even on Tablet or PC) that involve letters?”] and two to informal activities (e.g., “How often do you and your child sing nursery rhymes?”; “How often do you and your child read books or tell stories?”). Then, there were four questions on home numeracy activities, two related to direct (formal) activities [“How often do you and your child read or write numbers”; “How often do you and your child use games (even on Tablet or PC) that involve numbers”?] and two related to indirect (informal) activities [“How often do you and your child count objects?”; “How often do you and your child do simple calculations (2 + 1 = 3) in games or during other daily activities?”]. Responses were on a five-point Likert scale, from “never” to “everyday.” Sums of scores of each subscale (home literacy and home numeracy) were used in the analyses. Maximum score for each subscale was 20. Reliability for each scale was sufficient for the present sample (Home numeracy: Cronbach’s alpha = 0.69; Home literacy: Cronbach’s alpha = 0.67).

### Children’s Measures for the First Wave of Assessment (Second Year of Preschool)

#### Language Skills

The following tasks taken from the Learning Difficulties Indexes (IDA; Bonifacci et al., 2015) were used in the present study:

1. Vocabulary. Children were asked to name 36 images disposed on three grids with 12 images each selected for decreasing frequency in spoken language (Burani et al., 2001). The accuracy score, ranging from 0 to 36 (1 point for each correct answer), was considered. The Cronbach’s alpha of the scale was 0.85, according to the test manual.
2. Morpho-syntactic comprehension. Children were presented with three pictures representing three different scenarios. For each picture, they were asked to identify or manipulate elements of the scene by comprehending different types of sentences pronounced by the examiner (e.g., the child had to correctly place a card depicting a book after hearing a sentence such as “The book is under the pillow”). The morpho-syntactic structures investigated were: singular/plurals, locatives, active/passive, and relative clauses. A total of 18 sentences were presented, and for each of them, a score of 2 (correct answer at first attempt), 1 (correct answer at the second attempt), or 0 (wrong answer) was given. The total score, ranging from 0 to 36, was considered. The scale’s Cronbach’s alpha was 0.70, according to the test manual.

#### Non-symbolic Numeracy Skills

The tasks used were taken from the battery Number Sense: Prerequisites (Tobia et al., 2018), which assesses early numeracy skills in preschoolers.

1. Quantity comparison. Children were shown two illustrated baskets and were asked to quickly choose the one with a greater number of fruits in it, without counting, therefore relying on estimation processes. The number of fruits varied from 3 to 20, and the difference in quantity between sets ranged from 1 to 6 units. A total of 12 items was presented. A score of 1 (correct answer) or 0 (wrong answer) was given for each item, for a maximum total score of 12. There was a Cronbach’s alpha of 0.64, according to the test manual.
2. Seriation. This subtest included two tasks: (a) First, children were asked to put in ascending order a set of four pictures of the same object drawn in different dimensions (seriation with perceptual cues); (b) second, a fifth picture was given to the child, who was asked to put it in the correct place in the ordered composition (insertion). For each object placed in the correct position, a score of 1 was assigned. The total score, ranging from 0 to 20, was considered. The size seriation subtest’s KR-20 is 0.89, according to the test manual.

### Children’s Measures for the Second Wave of Assessment

#### Early Literacy Skills

1. Phonological awareness. This task was taken from the IDA battery (Bonifacci et al., 2015). It was a task of first syllable recognition (4 items). Children were given the image of an object (dog, bubble, sea, and pear) and four images amongst which the child was required to recognize the one whose name begins with the same sound [e.g., *cane* (dog), and *casa* (house)]. Each item received a score of 1 for correct responses and a score of 0 for incorrect answers, for a maximum total score of 4. The reliability score (KR-20) was 0.78, according to the test manual.
2. Letter knowledge. This task was adapted from the IDA battery (Bonifacci et al., 2015). Children were presented with a picture of a train with one letter (from a to z) in each coach. The experimenter said the sound of five letters (two vowels and three consonants), and the child was required to mark the correct letters on the sheet. A score of 1 was given for each correct response for a maximum score of 5. The Cronbach’s alpha of the scale calculated on the study’s sample was 0.77.
3. Early writing. This task was developed for the purpose of the present study. Children were asked to pretend to be writers, and they were asked to write five words: their first name, *ape* (bee), *serpente* (snake), *coccinella* (ladybug), and *treno* (train). A score from 0 (absence of signs) to 9 (all letters are correct and in proper order) was given for each word according to how the writing approximates the correct writing of the word. Scores ranged from 0 to 45, and Cronbach alpha calculated on the study’s sample was 0.87.

#### Symbolic Numeracy Skills

For being administered collectively, these tasks were adapted from the battery Number Sense: Prerequisites (Tobia et al., 2018).

1. Number recognition: Children receive a card with the digits 1 to 9 randomly distributed on a grid among blank squares. It is similar to a bingo card. Children are required to sign the number read aloud by the experimenter with a different colored pencil for each number. The examiner named five different numbers, and the score ranged from 0 to 5. Cronbach alpha was 0.89, according to the test manual.
2. Biunivocal correspondence. Children were provided with a card similar to the previous task, but boxes represented sets of elements (little stars ranging from 1 to 9). The examiner named five different numbers, requesting the child choose the set with the corresponding number of stars. For each digit correctly associated with a quantity, a score of 1 was given (score range: 0–5). Cronbach alpha was 0.77, according to the test manual.

### Procedure

Questionnaires on SES and home literacy/numeracy were given to parents during the first wave of assessment. Tasks in the first wave of assessment were administered individually by trained psychologists in a quiet room at the children’s school, in a single session lasting about 30 min. In the second wave, tasks were administered collectively in small groups of around 10–12 children in a single session lasting about 30 min. Breaks were allowed if the child showed signs of fatigue. Special attention was given to ascertaining that children had correctly understood the instructions; verbal instructions were minimized, and examples for each task were provided.

### Data Analysis

Preliminary analysis on outliers evidenced that few participants scored over the absolute value of 3 SDs on some tasks. These were less than 5% of the data, and we were allowed to proceed with the Winsorizing method (Duan, 1997; Wilcox, 2010), which suggests modifying outliers at the end of the tails of the distribution to the highest/lowest value within the distribution that are not suspected to be outliers. Then we checked the distribution, and due to a high level of negative skewness for some variables, we used the ln-transformation on these variables. The normality of the data improved and finally resulted normally distributed, particularly with skewness and kurtosis ranging in the limits of ±2 (acceptable values according to Trochim and Donnelly, 2006); these values are now reported in Table 1.

**TABLE 1:** Descriptive statistics of measures included in the study.

	Measure	Mean	SD	Min–max	Skewness	Kurtosis
First wave	Vocabulary^*^	32.42	2.54	11–36	0.48	0.17
	Morpho-syntactic comprehension^*^	29.46	4.37	2–36	0.21	−0.49
	Quantity comparison^*^	10.10	1.46	0–12	0.46	−0.56
	Seriation^*^	15.46	4.73	0–20	−0.3	−1.13
Second wave	Letter knowledge^*^	3.91	1.47	0–5	−0.64	−1.10
	Phonological awareness^*^	3.59	0.88	0–4	−1,53	0.85
	Early writing^*^	33.68	10.96	6–45	−0,15	−1.27
	Number recognition^*^	4.67	0.86	0–5	−1.92	1.95
	Biunivocal correspondence^*^	4.72	0.61	0–5	−1.49	0.45
Parents	Children’s SES^*^	47.04	10.60	13.5–61	0.10	−0.93
	Home Literacy	11.73	3.28	5–20	0.14	−0.6
	Home Numeracy	10.63	2.95	5–19	0.34	−0.37

^*^Skewness and kurtosis for ln-transformed values.

We did not find any issues of non-linear relationships between dependent and independent variables using the scatter plot graphic builder in SPSS v26. We also checked the plot of the standardized residuals errors by the regression standardized predicted values and found that all the residuals were distributed randomly around zero, meeting the homoscedasticity in our data.

Therefore, we concluded that parametric tests were suitable for these data, also considering the increased chance of Type II error when applying non-parametric analysis to (close to) normally distributed data (e.g., Hodges and Lehmann, 1956).

Pearson correlations between the main variables included in the study were performed.

A structural equation model (SEM; e.g., Kline, 2010), including CFA and path analysis, was applied using Amos software version 26.0 (Arbuckle, 2016) after transforming the variables into standardized scores. In this model, four latent variables were identified: Language, Early Literacy, Non-symbolic Numeracy, and Symbolic Numeracy, which include respectively: (1) vocabulary and morpho-syntactic comprehension, (2) early writing, letter knowledge and phonological awareness, (3) quantity comparison, size seriation, and (4) number recognition and biunivocal correspondence. A path analysis was used to examine the relationship between these latent dependent variables and SES as the independent variable through possible mediation of Home Literacy and Home Numeracy variables.

We also included directional paths from language to literacy and from non-symbolic to symbolic numeracy skills. This choice was supported either by the longitudinal design and by previous research that supported these developmental pathways for language to literacy (Foorman et al., 2015; Hulme et al., 2015) and from non-symbolic to symbolic (von Aster and Shalev, 2007).

The second model provides the same four latent variables, but in this case, the independent variables were Home Literacy and Home Numeracy, and we included the Home Literacy × Home Numeracy interaction. The SES variable was included as a mediator between the independent and dependent variables.

The SEM, including CFA, was run using Maximum Likelihood as the estimator method; for testing the mediation patterns, the Specific Indirect Effect Amos plugin was used (Gaskin et al., 2020). In order to reach a good fit, some adjustments were made following the suggestion of modification indexes without changing the key structure of the models (Kenny, 2011).

Multiple indices were used to evaluate models’ fit: Chi-square test of model fit (*χ*^2^); Root Mean Square Error of Approximation (RMSEA), Comparative Fit Index (CFI), Tucker-Lewis Index (TLI). A non-significant Chi-square test of model fit, TLI and CFI values equal to or higher than 0.90 indicate an acceptable model fit; RMSEA close to 0.08 or lower, indicate an acceptable fit (Marsh et al., 1988; Browne and Cudeck, 1993; Hu and Bentler, 1999). Cut-off values for both the RMSEA (0.01, 0.05, 0.08, and 0.10) and the CFI/TLI (0.99, 0.95, 0.92, and 0.90) have been commonly used to distinguish between excellent, close, fair, and mediocre or poor models, respectively (Hu and Bentler, 1999). In our study, the models’ indexes suggest a close fit to the data (see Table 3). Considering the new approach of the equivalence testing (Yuan et al., 2016), we interpreted our model fit indices more carefully. We can say that our models are sufficiently acceptable for describing our data.

**TABLE 2:** Pearson correlations coefficients (*r*) between SES, home literacy and numeracy, and children’s skills.

	SES	Home Literacy	Home Numeracy	Vocabulary	Morpho-syntactic comprehension	Quantity comparison	Seriation	Early writing	Letter knowledge	Phonological awareness	Number recognition
Home Literacy	0.175^**^										
Home Numeracy	0.139^*^	0.568^**^									
Vocabulary	0.129^*^	0.106	0,089								
Morpho-syntactic comprehension	0.182^**^	0.215^**^	0.214^**^	0.385^**^							
Quantity comparison	0.072	0.094	0.137^*^	0.183^**^	0.190^**^						
Seriation	0.173^**^	0.109	0.159^**^	0.173^**^	0.377^**^	0.166^**^					
Early writing	0.231^**^	0.289^**^	0.259^**^	0.178^**^	0.161^**^	0.001	0.129^*^				
Letter knowledge	0.193^**^	0.144^*^	0.138^*^	0.241^**^	0.188^**^	0.042	0.125^*^	0.345^**^			
Phonological awareness	0.196^**^	0.263^**^	0.240^**^	0.228^**^	0.208^**^	0.017	0.166^**^	0.512^**^	0.355^**^		
Number recognition	0.138^*^	0.077	0.254^**^	0.051	0.151^**^	−0.032	0.148^**^	0.318^**^	0.293^**^	0.393^**^	
Biunivocal correspondence	0.073	0.130^*^	0.114^*^	0.125^*^	0.131^*^	0.135^*^	0.107	0.224^**^	0.225^**^	0.304^**^	0.270^**^

^**^p < 0.01;

^*^p < 0.05 (two-tailed).

**TABLE 3:** Models’ parameters and statistics.

	Indirect path	Unstandardized estimation	CI lower	CI upper	*p*	*χ* ^2^	df	*p*	CFI	TLI	RMSEA
Model 1	SES → HomeLiteracy → Language	0.025	0.011	0.053	0.005	54.2	42.0	0.098	0.979	0.967	0.031
	SES → HomeLiteracy → Literacy	0.036	0.015	0.061	0.01						
	SES → HomeNumeracy → Non-symbolic number	0.019	0.004	0.046	0.017						
	SES → HomeNumeracy → Symbolic number	0.018	0.002	0.042	0.031						
Model 2	HomeLiteracy → SES → Language	0.002	0.001	0.004	0.032	61.0	50.0	0.137	0.982	0.971	0.027
	HomeLiteracy → SES → Literacy	0.002	0.001	0.006	0.026						
	HomeNumeracy → SES → Non-symbolic number	0.001	−0.001	0.004	0.258						
	HomeNumeracy → SES → Symbolic number	0.000	0.000	0.002	0.322						
	Home Literacy × Home Numeracy → Symbolic Numeracy				0.007						

## Results

Descriptive statistics and correlations among the observed variables are reported in Tables 1, 2, respectively. SES was significantly related to both home literacy and numeracy and with all measures in the linguistic domain. It was also related to numeracy skills, except biunivocal correspondence and quantity comparison. Home literacy and numeracy were significantly related between each other [*r*(308) = 0.568, *p* < 0.01], although not overlapping. Home literacy was further related to all language and literacy measures except vocabulary and, although to a lesser degree, with numeracy task of biunivocal correspondence. Home numeracy was related to all measures in the numeracy domain and with all language measures except vocabulary. Then, there were significant intra-domain relationships for all language measures. For numeracy measures, there were significant relationships between seriation and number recognition and between quantity comparison and biunivocal correspondence but not between quantity comparison and number recognition and between seriation and biunivocal correspondence. Considering inter-domain relationships, the highest correlations index was found between phonological awareness and number recognition [*r*(308) = 0.393, *p* < 0.01].

**FIGURE 1:**
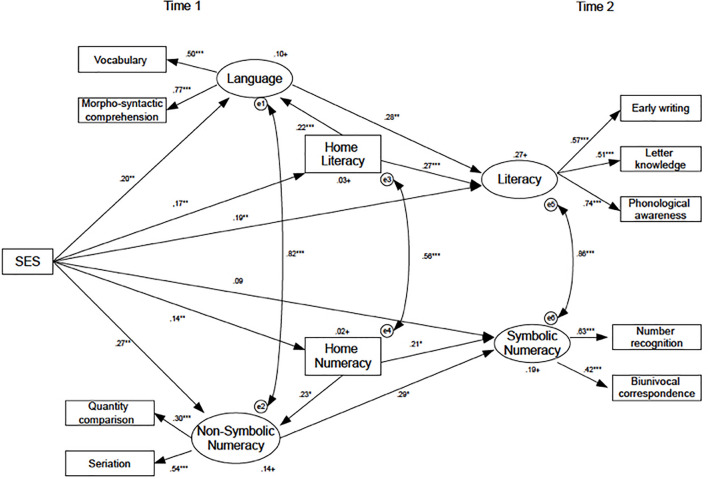
Model 1 on the relationships between SES and children’s early language/literacy and symbolic/non-symbolic numeracy skills with Home Literacy and Home Numeracy as mediators.

To better understand the predictive power of SES on children’s early literacy and numeracy skills and on home literacy and numeracy, a SEM was performed (Figure 1), which included home literacy and home numeracy as potential mediators. The SEM’s fit indexes were all acceptable (see Table 3).

The hypothesized path from the observed variable (SES) to the latent variables Language, Literacy, and Non-symbolic number was significant (*p* < 0.01), but the path from SES to Symbolic number was not (*p* > 0.05). All the other paths in the model were significant; see Figure 1 for the summary.

The mediation effect from SES and latent variables by Home Literacy and Home Numeracy were all significantly different from zero (see Table 3 for the results), concluding that Home Numeracy and Home literacy have a significant mediation effect in the relationships between SES and children’s skills. In particular, we have a partial mediation over Language, Literacy, and Non-symbolic numeracy and a full mediation over the Symbolic numeracy due to the significance of the indirect path only. Finally, language skills at age four predicted early literacy skills at age five (*p* < 0.01), and non-symbolic numeracy predicted symbolic numeracy from age four to age five (*p* < 0.05). All the other paths were significant, and the model’s fit was acceptable (see Table 3).

### Model 2

**FIGURE 2:**
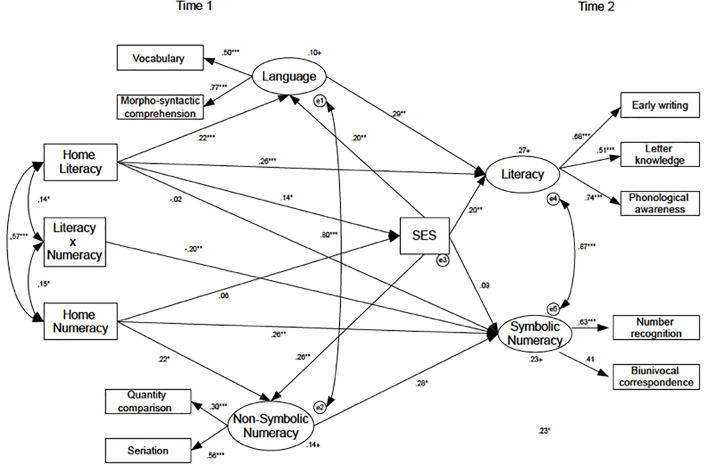
Model 2 on the Home Literacy × Home Numeracy interaction on children’s early language/literacy and symbolic/non-symbolic numeracy skills with SES as mediator.

For testing the interaction effects between Home literacy and Home numeracy on the latent variables referred to children’s skills (see Figure 2), we first standardized the scores and then used them in the models. In this case, we included SES as a mediator between the aforementioned variables. The SEM’s fit indexes were all acceptable (see Table 3).

**FIGURE 3:**
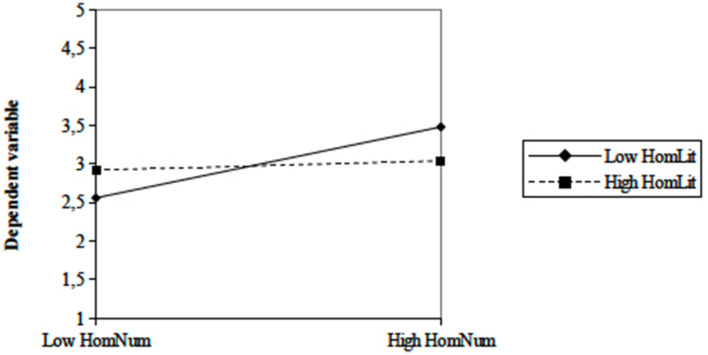
Interaction effect of home literacy and home numeracy on symbolic numeracy.

Results showed that the interaction effect is significant only on Symbolic-Numeracy (negative interaction, *p* < 0.01) and, therefore, the others interaction’s paths were deleted from the model. The interaction effect of home literacy and home numeracy on symbolic numeracy is shown in Figure 3 for a better understanding. The paths from Home Numeracy to SES and from SES to Symbolic Number were not significant (*p* > 0.05). All the other paths were significant; see the summary in Figure 2. Concerning the mediating role of SES, we found a significant mediation effect for both Early Literacy and Language, but we did not find a significant mediation effect on the numeracy skills.

## Discussion

In the present study, we first aimed to evaluate if SES was a direct predictor of children’s early language and literacy skills and symbolic and non-symbolic numeracy skills. We also tested if SES had a direct relationship with home literacy and numeracy and if these variables could mediate the relationship between SES and children’s skills. Secondly, we tested if home literacy and numeracy directly predicted children’s early literacy and numeracy skills. Finally, we evaluated the interaction’s effect of home literacy and numeracy on children’s skills through SES as a mediator. Importantly, this was a longitudinal study that included two waves of assessment, the first when children were in their second year of preschool and the second when they were in their final (third) year of preschool. SES, home literacy, and home numeracy were collected during the first wave of assessment.

We first explored correlations between the measures included in the study, and it emerged that SES was significantly related to all measures excluded biunivocal correspondence and quantity comparison. Then, there were domain-specific relationships between home literacy and language and literacy measures (except for a non-significant correlation between home literacy and vocabulary) and between home numeracy and all numeracy skills. Cross-domain relationships were found between home numeracy and all measures of language skills, but home literacy was related only with the numeracy task of biunivocal correspondence. Intra-domain relationships were found for all language measures but for the numeracy domain, there were only significant relationships between seriation and number recognition and between quantity comparison and biunivocal correspondence. There were also multiple cross-domain relationships amongst children’s skills, with the highest value for the correlation between phonological awareness and number knowledge.

Taken together, these results suggest a complex pattern of relationships that reinforce the evidence reported in the literature about reciprocal interactions between SES, home literacy and numeracy, and children’s early skills as well as cross-domain relationships between literacy and numeracy (Bonifacci et al., 2016; Cirino et al., 2018; Koponen et al., 2020; Bernabini et al., 2020a, 2021).

However, to better understand longitudinal causal pathways and cross-domain interactions, we developed two different SEM models.

In the first model, we considered SES to be a potential predictor of early language skills and early literacy skills, and symbolic and non-symbolic numeracy skills, including home literacy and numeracy as potential mediators. Results showed that SES had significant direct effects on early language and literacy skills. Home literacy significantly mediated the role of SES for language skills in the second year of preschool and early literacy skills at the end of preschool. Language skills were predictive of early literacy skills 1 year later. Concerning numeracy, it emerged that SES was predictive of non-symbolic skills but not of later symbolic skills. Home numeracy significantly mediated the role of SES on both waves of assessment. Also, early non-symbolic skills were significantly related to later numeracy skills. Considering the amount of variance explained in the model, this was higher for the second wave of assessment compared to the first and for early literacy (0.27) compared to symbolic numeracy (0.19).

This pattern of results reinforces some aspects of previous evidence and offers partially divergent results and new insights.

First of all, given the observed direct path from home literacy and numeracy to children’s skills, this study reinforces the body of evidence that highlights the importance of home literacy on children’s early language and literacy skills (e.g., Sénéchal et al., 1998) and that of home numeracy on early numeracy skills (e.g., Skwarchuk et al., 2014). Secondly, our results are in line with previous evidence of a direct relationship between SES and early language skills (Hoff, 2003). Although there was less evidence in this regard, we found that SES was also related to early literacy skills. Considering the relationship between home literacy and early language skills, these results diverge from those found in the Italian context by Bonifacci et al. (2021), where no direct path was observed from SES and home literacy to early literacy skills. The two studies, however, differ in some critical aspects. In Bonifacci et al. (2021), the authors also included measures of executive functions (inhibition and working memory) that may have dampened the influence of SES. Also, the present study involved a larger sample and considered home literacy and numeracy as mediators rather than as independent variables. However, it has to be underlined that also in the present study, in line with Bonifacci et al. (2021) and different from previous evidence (e.g., Hoff, 2013), we did not find, at a correlational level, significant relationships between SES and children’s vocabulary. Therefore, it might be that some cultural differences between Italian and American/Canadian mothers (Richman et al., 1988; Girolametto et al., 2002; Hsu and Lavelli, 2005) intervene in the relationship between SES and early linguistic skills. We might suggest that more research is needed in different cultural contexts to better understand the stability of these relationships and the factors that might intervene.

Considering the relationship between SES and the numeracy domain, our results found that SES was significantly related to non-symbolic but not symbolic skills. These results contrast with some previous studies (Jordan and Levine, 2009), which found that SES disparities were differently related to subcomponents of numeracy skills, with higher gaps in the symbolic tasks and minor or no differences in performance in non-symbolic tasks. A reversed pattern was observed in the present study, with SES being related to non-symbolic skills but not to symbolic skills. Future studies will need to address this issue with more comprehensive measures of symbolic and non-symbolic skills. A potential explanation to the present pattern of results is linked to the hypotheses that parents with low SES might have had previous math difficulties and that intergenerational patterns might mediate the relationship between low-SES and early non-symbolic skills. In this regard, there is some evidence of intergenerational pathways in non-symbolic numeracy skills (Braham and Libertus, 2017; Navarro et al., 2018; Bernabini et al., 2020b). Since the absence of relationships of SES and symbolic numeracy skills, it might be that the school context might act as a protective factor. If children are exposed to proper early numeracy activities at school, this might reduce the impact of SES on symbolic numeracy skills. Future investigations should consider the quality and quantity of school activities in these domains.

Significantly, this study adds important new insights with respect to previous literature showing that, although SES predicted both home literacy and numeracy skills, home literacy partially mediates the effect of SES on language and literacy measures in addition to non-symbolic numeracy and fully mediates the relationship between SES and symbolic numeracy skills. Previous studies already suggested an influence of SES on home literacy (van Steensel, 2006) and home numeracy (DeFlorio and Beliakoff, 2015), although others reported opposite patterns, with more home activities in low-SES parents (Silinskas et al., 2010) or no effects of SES on home literacy and numeracy (de Keyser et al., 2020). To our knowledge, no previous study evaluated these mediation effects considering together different components of early language, literacy, and symbolic and non-symbolic aspects of numeracy skills. We found evidence that home literacy and home numeracy mediate the relationship between SES and children’s skills. A previous study found similar results on first-grade children, but it focused on single measures of reading and math achievement and only considered mother’s education as a proxy of SES (Zadeh et al., 2010). Also, in line with our study Galindo and Sonnenschein (2015) found that home numeracy mediated the relationship between SES and math skills. We, therefore, can conclude that all aspects of the home environment mediated to a certain degree the associations between SES and children’s skills and that home environment during the preschool years might reduce the detrimental effects of low maternal education on children’s ability, for both literacy and numeracy skills and particularly for symbolic numeracy skills.

As a second aim of the study, we also wanted to understand the cross-domain effects of home literacy and numeracy and children’s skills. To this purpose, we developed a model of the interaction between home literacy and numeracy on the two different domains, including SES as a mediator. It emerged that the interaction of home literacy and home numeracy was not significant in language and literacy skills and non-symbolic numeracy measures. Instead, it was negatively significant for children’s symbolic numeracy skills. The analysis revealed that if home literacy is high when home numeracy is low, this enhances numeracy skills (mainly symbolic ones). These results are partially in line with previous studies that found a relationship between home literacy and numeracy skills (LeFevre et al., 2009, 2010; Anders et al., 2012; Soto-Calvo et al., 2020) and reinforce the role of language on numeracy skills (Hauser et al., 2010). It contradicts previous evidence about the possibility that home numeracy predicts language and literacy skills (Huntsinger et al., 2016; Napoli and Purpura, 2018). This is a novel contribution of the present study since, to our knowledge, no previous study directly addressed this issue, considering literacy and numeracy skills considered at different time moments. Finally, Model 2 reinforces and enriches findings from Model 1 regarding the role of SES. In Model 2, SES was included as a mediator of the relationship between home literacy and numeracy, and children’s skills. It was found that SES significantly mediates the role of home literacy on early language and literacy skills but not that of home numeracy on children’s numeracy skills. These results suggest that SES might have a more prominent role in the language and literacy domains compared to the numeracy domain (Silinskas et al., 2010; Baird, 2012; de Keyser et al., 2020).

There are some limitations of the present study that need to be considered. First, we did not test specific relationships of SES and home literacy and numeracy with single literacy and numeracy factors, although this was partially considered in correlation analyses. In other words, it might be that SES might differently affect subdomains of literacy and numeracy, and this should be considered in forthcoming studies. In addition, measures of home literacy and numeracy did not distinguish between formal and informal activities. We opted for a short questionnaire to encourage greater adherence to the study, proposing a questionnaire that is easy to fill out by parents and in line with other studies that adopted a similar approach (Stephenson et al., 2008; Manolitsis et al., 2013). However, the absence of information about the differential role of formal and informal home literacy and numeracy activities might limit the generalizability of results and would require further investigation. Finally, the study was conducted on Italian monolingual children who showed considerable variation in SES scores but could not be considered a low-SES sample. Other studies should be performed on low-SES samples and on children from a migrant background where bilingual exposure in the home and family environment might differently interact with SES, home literacy and numeracy variables, and children’s skills (Bonifacci et al., 2020).

Despite these limitations, the present study adds a significant contribution to the previous evidence regarding three main points. First, the study evidenced that home literacy and home numeracy partially or fully mediated the relationships between SES and children’s skills, suggesting that home activities might dampen the detrimental effects of SES on children’s skills. Secondly, the study highlighted a significant interaction of home literacy and home numeracy on symbolic numeracy skills, suggesting that home literacy might represent a protective factor when home numeracy is low. Finally, the present study was conducted on monolingual Italian children and their parents; Italian is a highly transparent language, and, since most studies were conducted on opaque language such as English, the study adds insights about the generalizability of results of previous studies to different linguistic and cultural contexts.

These results have potential implications for educational settings. Given the potential role of home literacy and home numeracy in mediating children’s literacy and numeracy skills, the present study indirectly reinforces the importance of implementing parents’ intervention programs to foster home literacy and numeracy practices. These interventions might reduce the negative impact of SES on children’s early literacy and numeracy skills and possibly on future academic achievements. Particular attention should be given to low-SES populations for whom intervention programs might be of specific relevance and impact. This study also suggests that parents’ intervention programs should focus on both literacy and numeracy activities.

## Data Availability Statement

The raw data supporting the conclusions of this article will be made available by the authors, without undue reservation.

## Ethics Statement

The studies involving human participants were reviewed and approved by the Bioethics Committee of the University of Bologna. Written informed consent to participate in this study was provided by the participants’ legal guardian/next of kin.

## Author Contributions

BP and PB conceived the presented idea, developed the assessment protocol, and wrote a preliminary version of the manuscript. BP carried out the experiment and collected and analyzed the data. DC and PB performed the statistical analyses. AF and BP contributed to the interpretation of the results. AF, BP, and PB revised and significantly contributed to the final version of the manuscript. All authors discussed the results and contributed to the final manuscript.

## Conflict of Interest

The authors declare that the research was conducted in the absence of any commercial or financial relationships that could be construed as a potential conflict of interest.

## Publisher’s Note

All claims expressed in this article are solely those of the authors and do not necessarily represent those of their affiliated organizations, or those of the publisher, the editors and the reviewers. Any product that may be evaluated in this article, or claim that may be made by its manufacturer, is not guaranteed or endorsed by the publisher.

## References

[B1] Adler N. E. Newman K. (2002). Socio-economic disparities in health: pathways and policies. *Health Affairs* 21 60–76. 10.1377/hlthaff.21.2.60 11900187

[B2] Anders Y. Rossbach H. G. Weinert S. Ebert S. Kuger S. Lehrl S. (2012). Home and preschool learning environments and their relations to the development of early numeracy skills. *Early Child. Res. Q.* 27 231–244. 10.1016/j.ecresq.2011.08.003

[B3] Arbuckle J. L. (2016). *Amos (Version 26.0) [Computer Program].* Chicago: IBM SPSS.

[B4] Baird K. (2012). Class in the classroom: The relationship between school resources and math performance among low socio-economic status students in 19 rich countries. *Educ. Eco.* 20 484–509. 10.1080/09645292.2010.511848

[B5] Baker C. E. (2014). Mexican mothers’ English proficiency and children’s school readiness: mediation through home literacy involvement. *Early Educ. Dev.* 25 338–355. 10.1080/10409289.2013.807721

[B6] Bellocchi S. Tobia V. Bonifacci P. (2017). Predictors of reading and comprehension abilities in bilingual and monolingual children: A longitudinal study on a transparent language. *Read. Writ.* 30 1311–1334.

[B7] Benzies K. Mychasiuk R. (2009). Fostering family resiliency: A review of the key protective factors. *Child Family Soc. Work* 14 103–114. 10.1111/j.1365-2206.2008.00586.x

[B8] Bernabini L. Bonifacci P. de Jong P. F. (2021). The relationship of reading abilities with the underlying cognitive skills of math: a dimensional approach. *Front. Psychol.* 12:577488. 10.3389/fpsyg.2021.577488 33716850 PMC7946841

[B10] Bernabini L. Tobia V. Guarini A. Bonifacci P. (2020b). Predictors of children’s early numeracy: environmental variables, intergenerational pathways, and children’s cognitive, linguistic, and non-symbolic number skills. *Front. Psychol.* 11:505065. 10.3389/fpsyg.2020.505065 33240141 PMC7677194

[B9] Bernabini L. Tobia V. Bonifacci P. (2020a). Intergenerational features of math skills: symbolic and non-symbolic magnitude comparison and written calculation in mothers and children. *J. Cogn. Dev.* 22 149–167. 10.1080/15248372.2020.1844711

[B11] Blevins-Knabe B. Austin A. B. Musun L. Eddy A. Jones R. M. (2000). Family home care providers’ and parents’ beliefs and practices concerning mathematics with young children. *Early Child Dev. Care* 165 41–58. 10.1080/0300443001650104

[B12] Blevins-Knabe B. Musun-Miller L. (1996). Number use at home by children and their parents and its relationship to early mathematical performance. *Infant Child Dev.* 5 35–45. 10.1002/(sici)1099-0917(199603)5:1<35::aid-edp113>3.0.co;2-0

[B13] Bollen K. A. Glanville J. L. Stecklov G. (2001). Socio-economic status and class in studies of fertility and health in developing countries. *Annu. Rev. Sociol.* 27 153–185. 10.1146/annurev.soc.27.1.153

[B14] Bonifacci P. Lombardo G. Pedrinazzi J. Terracina F. Palladino P. (2020). Literacy skills in bilinguals and monolinguals with different SES. *Read. Writ. Q.* 36 243–259. 10.1080/10573569.2019.1635057

[B15] Bonifacci P. Pellizzari C. Giuliano P. Serra P. (2015). *IDA - Indicatori delle Difficoltà di Apprendimento.* Firenze: Hogrefe Editore.

[B16] Bonifacci P. Tobia V. Bernabini L. Marzocchi G. M. (2016). Early literacy and numeracy skills in bilingual minority children: Toward a relative independence of linguistic and numerical processing. *Front. Psychol.* 7:1020. 10.3389/fpsyg.2016.01020 27458413 PMC4935724

[B17] Bonifacci P. Trambagioli N. Bernabini L. Tobia V. (2021). Home activities and cognitive skills in relation to early literacy and numeracy: Testing a multifactorial model in preschoolers. *Eur. J. Psychol. Educ.* 10.1007/s10212-021-00528-2 [Epub ahead of print].

[B18] Bradley R. H. Corwyn R. F. (2002). Socio-economic status and child development. *Annu. Rev. Psychol.* 53 371–399. 11752490 10.1146/annurev.psych.53.100901.135233

[B19] Braham E. J. Libertus M. E. (2017). Intergenerational associations in numerical approximation and mathematical abilities. *Dev. Sci.* 20:desc.12436. 10.1111/desc.12436 27496658 PMC6035787

[B20] Braham E. J. Libertus M. E. McCrink K. (2018). Children’s spontaneous focus on number before and after guided parent–child interactions in a children’s museum. *Dev. Psychol.* 54:1492. 10.1037/dev0000534 30047774 PMC6132254

[B21] Brese F. Mirazchiyski P. (2013). *Measuring students’ family background in large-scale international education studies. Issues and methodologies in large-scale assessments. Special issue 2. IERI Monograph series.* Hamburg: IERI.

[B22] Broer M. Bai Y. Fonseca F. (2019). *Socio-economic inequality and educational outcomes: Evidence from twenty years of TIMSS.* Cham: Springer Open.

[B23] Browne M. W. Cudeck R. (1993). “Alternative ways of assessing model fit,” in *Testing Structural Equation Models* eds Bollen K. A. Long J. S. (Newbury Park, CA: Sage) 136–162.

[B24] Buckingham J. Beaman R. Wheldall K. (2014). Why poor children are more likely to become poor readers: The early years. *Educ. Rev.* 66 428–446.

[B25] Burani C. Barca L. Arduino L. S. (2001). Una base di dati sui valori di età di acquisizione, frequenza, familiarità, immaginabilità, concretezza, e altre variabili lessicali e sublessicali per 626 nomi dell’italiano [Dataset on age of acquisition, familiarity, imageability, concreteness, and other lexical and sublexical variables for 626 Italian words]. *G. Ital. Psicol.* 28 839–856.

[B26] Caravolas M. Lervåg A. Defior S. Seidlová Málková G. Hulme C. (2013). Different patterns, but equivalent predictors, of growth in reading in consistent and inconsistent orthographies. *Psychol. Sci.* 24 1398–1407. 10.1177/0956797612473122 23744876

[B27] Case A. Lubotsky D. Paxson C. (2002). Economic status and health in childhood: The origins of the gradient. *Am. Econ. Rev.* 92 1308–1334. 10.1257/000282802762024520 29058397

[B28] Cirino P. T. Child A. E. Macdonald K. T. (2018). Longitudinal predictors of the overlap between reading and math skills. *Contemp. Educ. Psychol.* 54 99–111. 10.1016/j.cedpsych.2018.06.002 30559576 PMC6294126

[B29] Clarke B. Shinn M. R. (2004). A preliminary investigation into the identification and development of early mathematics curriculum-based measurement. *School Psychol. Rev.* 33:234. 10.1080/02796015.2004.12086245

[B30] Davis-Kean P. E. (2005). The influence of parent education and family income on child achievement: The indirect role of parental expectations and the home environment. *J. Family Psychol.* 19 294–304. 10.1037/0893-3200.19.2.294 15982107

[B31] de Keyser L. Bakker M. Rathé S. Wijns N. Torbeyns J. Verschaffel L. (2020). No association between the home math environment and numerical and patterning skills in a large and diverse sample of 5-to 6-year-olds. *Front. Psychol.* 11:547626. 10.3389/fpsyg.2020.547626 33362620 PMC7758193

[B32] DeFlorio L. Beliakoff A. (2015). Socio-economic status and preschoolers’ mathematical knowledge: The contribution of home activities and parent beliefs. *Early Educ. Dev.* 26 319–341. 10.1080/10409289.2015.968239

[B33] Dehaene S. (1992). Varieties of numerical abilities. *Cognition* 44 1–42. 10.1016/0010-0277(92)90049-n 1511583

[B34] Dehaene S. (2001). Précis of the number sense. *Mind Lang.* 16 16–36.

[B35] Duan B. (1997). *The robustness of trimming and Winsorization when the population distribution is skewed*. Unpublished dissertation. New Orleans: Tulane University.

[B36] Duncan G. J. Magnuson K. (2011). The nature and impact of early achievement skills, attention skills, and behavior problems. *Whither Opport.* 2011 47–70.

[B37] Ehri L. C. Nunes S. R. Willows D. M. Schuster B. V. Yaghoub-Zadeh Z. Shanahan T. (2001). Phonemic awareness instruction helps children learn to read: evidence from the national reading Panel’s meta-analysis. *Read. Res. Q.* 36 250–287. 10.1598/rrq.36.3.2

[B38] Elliott L. (2020). Sources of heterogeneity in the home learning environments of socioeconomically disadvantaged families. *J. Appl. Dev. Psychol.* 70:10 1190.

[B39] Elliott L. Bachman H. J. (2018). SES disparities in early math abilities: The contributions of parents’ math cognitions, practices to support math, and math talk. *Dev. Rev.* 49 1–15. 10.1016/j.dr.2018.08.001

[B40] Elliott L. Braham E. J. Libertus M. E. (2017). Understanding sources of individual variability in parents’ number talk with young children. *J. Exp. Child Psychol.* 159 1–15. 10.1016/j.jecp.2017.01.011 28266331

[B41] Evans M. A. Shaw D. Bell M. (2000). Home literacy activities and their influence on early literacy skills. *Can. J. Exp. Psychol.* 54 65–75. 10881391 10.1037/h0087330

[B42] Foorman B. R. Herrera S. Petscher Y. Mitchell A. Truckenmiller A. (2015). The structure of oral language and reading and their relation to comprehension in Kindergarten through Grade 2. *Read. Writ.* 28 65 5–681. 10.1007/s11145-015-9544-5 PMC5029469 27660395

[B43] Foy J. Mann V. (2003). Home literacy environment and phonological awareness in preschool children: Differential effects for rhyme and phoneme awareness. *Appl. Psychol.* 24 59–88. 10.1017/s0142716403000043

[B44] Galindo C. Sonnenschein S. (2015). Decreasing the SES math achievement gap: Initial math proficiency and home learning environments. *Contemp. Educ. Psychol.* 43 25–38. 10.1016/j.cedpsych.2015.08.003

[B45] Gallistel C. R. Gelman R. (1992). Preverbal and verbal counting and computation. *Cognition* 44 43–74. 10.1016/0010-0277(92)90050-r 1511586

[B46] Gallistel C. R. Gelman R. (2000). Non-verbal numerical cognition: From reals to integers. *Trends Cogn. Sci.* 4 59–65. 10.1016/s1364-6613(99)01424-2 10652523

[B47] Gaskin J. James M. Lim J. (2020). *“Indirect Effects”, AMOS Plugin. Gaskination’s StatWiki.* Available online at: http://statwiki.gaskination.com/index.php?title=Plugins

[B48] Girolametto L. Bonifacio S. Visini C. Weitzman E. Zocconi E. Pearce P. (2002). Mother-child interactions in Canada and Italy: Linguistic responsiveness to late-talking toddlers. *Int. J. Lang. Commun. Disord.* 37 153–171. 10.1080/13682820110116794 12012613

[B49] Göbel S. M. Watson S. E. Lervåg A. Hulme C. (2014). Children’s arithmetic development: it is number knowledge, not the approximate number sense, that counts. *Psychol. Sci.* 25 789–798. 10.1177/0956797613516471 24482406

[B50] Guo Y. Puranik C. Kelcey B. Sun J. Dinnesen M. S. Breit-Smith A. (2020). The role of home literacy practices in kindergarten children’s early writing development: A one-year longitudinal study. *Early Educ. Dev.* 32 1–19. 10.1080/13670050.2021.1943304

[B51] Hannon P. Nutbrown C. Morgan A. (2020). Effects of extending disadvantaged families’ teaching of emergent literacy. *Res. Papers Educ.* 35 310–336. 10.1080/02671522.2019.1568531

[B52] Hauser M. Chomsky N. Fitch W. (2010). “The faculty of language: What is it, who has it, and how did it evolve?,” in *The Evolution of Human Language: Biolinguistic Perspectives*, eds Larson R. Déprez V. Yamakido H. (Cambridge: Cambridge University Press), 14–42. 10.1017/cbo9780511817755.002

[B53] Hecht S. A. Torgesen J. K. Wagner R. K. Rashotte C. A. (2001). The relations between phonological processing abilities and emerging individual differences in mathematical computation skills: A longitudinal study from second to fifth grades. *J. Exper. Child Psychol.* 79 19 2–227. 10.1006/jecp.2000.2586 11343408

[B54] Hemmerechts K. Agirdag O. Kavadias D. (2017). The relationship between parental literacy involvement, socio-economic status, and reading literacy. *Educ. Rev.* 69 85–101. 10.1080/00131911.2016.1164667

[B55] Hodges J. Lehmann E. L. (1956). The efficiency of some non-parametric competitors of the t-test. *Ann. Math. Stat.* 27 32 4–335.

[B56] Hoff E. (2003). “Causes and consequences of SES-related differences in parent-to-child speech,” in *Monographs in parenting series. Socio-economic status, parenting, and child development*, eds Bornstein M. H. Bradley R. H. (Mahwah, NJ: Lawrence Erlbaum Associates Publishers), 147–160.

[B57] Hoff E. (2013). Interpreting the early language trajectories of children from low-SES and language minority homes: Implications for closing achievement gaps. *Dev. Psychol.* 49:4. 10.1037/a0027238 22329382 PMC4061698

[B58] Hofslundsengen H. Gustafsson J. E. Hagtvet B. E. (2019). Contributions of the home literacy environment and underlying language skills to preschool invented writing. *Scandinavian J. Educ. Res.* 63 653–669. 10.1080/00313831.2017.1420686

[B59] Hollingshead A. B. (2011). Four factor index of social status. *Yale J. Sociol.* 8 21–51.

[B60] Hood M. Conlon E. Andrews G. (2008). Preschool home literacy practices and children’s literacy development: A longitudinal analysis. *J. Educ. Psychol.* 100:252. 10.1037/0022-0663.100.2.252

[B61] Hsu H. C. Lavelli M. (2005). Perceived and observed parenting behavior in American and Italian first time mothers across the first 3 months. *Infant Behav. Dev.* 28 503–518.

[B62] Hu L. T. Bentler P. M. (1999). Cutoff criteria for fit indexes in covariance structure analysis: Conventional criteria versus new alternatives. *Struc. Equation Model. Multidiscip. J.* 6 1–55. 10.1080/10705519909540118

[B63] Hulme C. Nash H. M. Gooch D. Lervåg A. Snowling M. J. (2015). The foundations of literacy development in children at familial risk of dyslexia. *Psychol. Sci.* 26 1877–1886. 10.1177/0956797615603702 26525072 PMC4676358

[B64] Huntley-Fenner G. Cannon E. (2000). Preschoolers’ magnitude comparisons are mediated by a preverbal analog mechanism. *Psychol. Sci.* 11 14 7–152. 10.1111/1467-9280.00230 11273422

[B65] Huntsinger C. S. Jose P. E. Luo Z. (2016). Parental facilitation of early mathematics and reading skills and knowledge through encouragement of home-based activities. *Early Child. Res. Q.* 37 1–15. 10.1016/j.ecresq.2016.02.005

[B66] Inoue T. Manolitsis G. de Jong P. F. Landerl K. Parrila R. Georgiou G. K. (2020). Home literacy environment and early literacy development across languages varying in orthographic consistency. *Front. Psychol.* 11:1923. 10.3389/fpsyg.2020.01923 32849130 PMC7412602

[B67] Jordan N. C. Levine S. C. (2009). Socio-economic variation, number competence, and mathematics learning difficulties in young children. *Dev. Disabil. Res. Rev.* 15 60–68. 10.1002/ddrr.46 19213011

[B68] Kenny D. A. (2011). *Respecification of latent variable models.* Available online at: http://davidakenny.net/cm/respec.htm (accessed January, 2021).

[B69] Kleemans T. Peeters M. Segers E. Verhoeven L. (2012). Child and home predictors of early numeracy skills in kindergarten. *Early Child. Res. Q.* 27 471–477. 10.1016/j.ecresq.2011.12.004

[B70] Kleemans T. Segers E. Verhoeven L. (2016). Relations between home numeracy experiences and basic calculation skills of children with and without specific language impairment. *Early Child. Res. Q.* 28 415–423.

[B71] Kline R. B. (2010). *Principles and Practice of Structural Equation Modeling* 3rd Edn. New York, NY: Guilford Press.

[B72] Koponen T. Eklund K. Heikkilä R. Salminen J. Fuchs L. Fuchs D. (2020). Cognitive correlates of the covariance in reading and arithmetic fluency: Importance of serial retrieval fluency. *Child Dev.* 91 1063–1080. 10.1111/cdev.13287 31292957

[B73] LeFevre J. A. Polyzoi E. Skwarchuk S. L. Fast L. Sowinski C. (2010). Do home numeracy and literacy practices of Greek and Canadian parents predict the numeracy skills of kindergarten children? *Int. J. Early Years Educ.* 18 55–70. 10.1080/09669761003693926

[B74] LeFevre J. A. Skwarchuk S. L. Smith-Chant B. L. Fast L. Kamawar D. Bisanz J. (2009). Home numeracy experiences and children’s math performance in the early school years. *Can. J. Behav. Sci.* 41:55. 10.1037/a0014532

[B75] Levine S. C. Jordan N. C. Huttenlocher J. (1992). Development of calculation abilities in young children. *J. Exper. Child Psychol.* 53 72–103. 10.1016/s0022-0965(05)80005-0 1545190

[B76] Leyva D. Tamis-LeMonda C. S. Yoshikawa H. Jimenez-Robbins C. Malachowski L. (2017). Grocery games: How ethnically diverse low-income mothers support children’s reading and mathematics. *Early Child. Res. Q.* 40 63–76. 10.1016/j.ecresq.2017.01.001

[B77] Linver M. R. Brooks-Gunn J. Kohen D. E. (2002). Family processes as pathways from income to young children’s development. *Dev. Psychol.* 38 719–734. 10.1037/0012-1649.38.5.719 12220050

[B78] Manolitsis G. Georgiou G. K. Tziraki N. (2013). Examining the effects of home literacy and numeracy environment on early reading and math acquisition. *Early Child. Res. Q.* 28 692–703. 10.1016/j.ecresq.2013.05.004

[B79] Marsh H. W. Balla J. R. McDonald R. P. (1988). Goodness-of-fit indexes in confirmatory factor analysis: The effect of sample size. *Psychol. Bull.* 103:391. 10.1037/0033-2909.103.3.391

[B80] Melhuish E. C. Phan M. B. Sylva K. Sammons P. Siraj-Blatchford I. Taggart B. (2008). Effects of the home learning environment and preschool center experience upon literacy and numeracy development in early primary school. *J. Soc. Issues* 64 95–114. 10.1111/j.1540-4560.2008.00550.x

[B81] Mistry R. S. Benner A. D. Biesanz J. C. Clark S. L. (2010). Family and social risk, and parental investments during the early childhood years as predictors of low-income children’s school readiness outcomes. *Early Child. Res. Q.* 25 432–449. 10.1016/j.ecresq.2010.01.002

[B82] Mutaf Yildiz B. Sasanguie D. De Smedt B. Reynvoet B. (2018). Investigating the relationship between two home numeracy measures: A questionnaire and observations during Lego building and book reading. *Br. J. Dev. Psychol.* 36 354–370. 10.1111/bjdp.12235 29393519

[B83] Mutaf- Yildiz B. Sasanguie D. De Smedt B. Reynvoet B. (2020). Probing the relationship between home numeracy and children’s mathematical skills: A systematic review. *Front. Psychol.* 11:2074. 33071838 10.3389/fpsyg.2020.02074 PMC7530373

[B84] Napoli A. R. Purpura D. J. (2018). The home literacy and numeracy environment in preschool: Cross-domain relations of parent-child practices and child outcomes. *J. Exper. Child Psychol.* 166 581–603. 10.1016/j.jecp.2017.10.002 29102840

[B85] Navarro M. G. Braham E. J. Libertus M. E. (2018). Intergenerational associations of the approximate number system in toddlers and their parents. *Br. J. Dev. Psychol.* 36 521–539. 10.1111/bjdp.12234 29377230

[B86] Niklas F. Schneider W. (2014). Casting the die before the die is cast: The importance of the home numeracy environment for preschool children. *Eur. J. Psychol. Educ.* 29 327–345. 10.1007/s10212-013-0201-6

[B87] Pan Y. Gauvain M. Liu Z. Cheng L. (2006). American and Chinese parental involvement in young children’s mathematics learning. *Cogn. Dev.* 21 17–35. 10.1016/j.cogdev.2005.08.001

[B88] Park H. (2008). Home literacy environments and children’s reading performance: A comparative study of 25 countries. *Educ. Res. Eval.* 14 489–505. 10.1080/13803610802576734

[B89] Payne A. C. Whitehurst G. J. Angell A. L. (1994). The role of home literacy environment in the development of language ability in preschool children from low-income families. *Early Child. Res. Q.* 9 427–440. 10.1016/0885-2006(94)90018-3

[B90] Puranik C. S. Phillips B. M. Lonigan C. J. Gibson E. (2018). Home literacy practices and preschool children’s emergent writing skills: An initial investigation. *Early Child. Res. Q.* 42 228–238. 10.1016/j.ecresq.2017.10.004

[B91] Purpura D. J. Logan J. A. R. (2015). The non-linear relations of the approximate number system and mathematical language to early mathematics development. *Dev. Psychol.* 51 1717–1724. 10.1037/dev0000055 26436871

[B92] Reardon S. F. Portilla X. A. (2016). Recent trends in income, racial, and ethnic school readiness gaps at kindergarten entry. *Aera Open* 2:233285841665734. 10.1177/2332858416657343

[B93] Reutzel D. R. Fawson P. C. Smith J. A. (2005). Words to go!: Evaluating a first-grade parent involvement program for “making” words at home. *Literacy Res. Instruc.* 45 119–159. 10.1080/19388070609558445

[B94] Richman A. L. LeVine R. A. New R. S. Howrigan G. A. Welles-Nystrom B. LeVine S. E. (1988). Maternal behavior to infants in five cultures. *New Dir. Child Dev.* 40 81–97. 10.1002/cd.23219884010 3217040

[B95] Roberts J. Jergens J. Burchinal M. (2005). The role of home literacy practices in preschool children’s language and emergent literacy skills. *J. Speech. Lang. Hear. Res.* 48 345–359. 10.1044/1092-4388(2005/024) 15989397

[B96] Saint-Laurent L. Giasson J. (2005). Effects of a family literacy program adapting parental intervention to first graders’ evolution of reading and writing abilities. *J. Early Child. Literacy* 5 253–278. 10.1177/1468798405058688

[B97] Sénéchal M. (2006). Testing the home literacy model: Parent involvement in kindergarten is differentially related to grade 4 reading comprehension, fluency, spelling, and reading for pleasure. *Sci. Stud. Read.* 10 59–87. 10.1207/s1532799xssr1001_4

[B98] Sénéchal M. LeFevre J. A. (2002). Parental involvement in the development of children’s reading skill: A five-year longitudinal study. *Child Dev.* 73 4 45–460. 10.1111/1467-8624.00417 11949902

[B99] Sénéchal M. LeFevre J. A. Thomas E. M. Daley K. E. (1998). Differential effects of home literacy experiences on the development of oral and written language. *Read. Res. Q.* 33 96–116. 10.1598/rrq.33.1.5

[B100] Sénéchal M. Whissell J. Bildfell A. (2017). Starting from home: Home literacy practices that make a difference. *Theor.Read. Dev.* 15:383.

[B101] Silinskas G. Leppänen U. Aunola K. Parrila R. Nurmi J. E. (2010). Predictors of mothers’ and fathers’ teaching of reading and mathematics during kindergarten and Grade 1. *Learn. Instruc.* 20 61–71. 10.1016/j.learninstruc.2009.01.002

[B102] Sim F. Thompson L. Marryat L. Ramparsad N. Wilson P. (2019). Predictive validity of preschool screening tools for language and behavioural difficulties: A PRISMA systematic review. *PloS One* 14:e0211409. 10.1371/journal.pone.0211409 30716083 PMC6361441

[B103] Sirin S. R. (2005). Socio-economic status and academic achievement: a metanalytic review of research. *Rev. Educ. Res.* 75 417–453. 10.3102/00346543075003417

[B104] Skwarchuk S. L. Sowinski C. LeFevre J. A. (2014). Formal and informal home learning activities in relation to children’s early numeracy and literacy skills: The development of a home numeracy model. *J. Exper. Child Psychol.* 121 63–84. 10.1016/j.jecp.2013.11.006 24462995

[B105] Soto-Calvo E. Simmons F. R. Adams A. M. Francis H. N. Patel H. Giofrè D. (2020). Identifying the preschool home learning experiences that predict early number skills: Evidence from a longitudinal study. *Early Child. Res. Q.* 53 314–328. 10.1016/j.ecresq.2020.04.004

[B106] Stephenson K. A. Parrila R. K. Georgiou G. K. Kirby J. R. (2008). Effects of home literacy, parents’ beliefs, and children’s task-focused behavior on emergent literacy and word reading skills. *Sci. Stud. Read.* 12 24–50.

[B107] Suggate S. Schaughency E. McAnally H. Reese E. (2018). From infancy to adolescence: The longitudinal links between vocabulary, early literacy skills, oral narrative, and reading comprehension. *Cogn. Dev.* 47 82–95. 10.1016/j.cogdev.2018.04.005

[B108] Tobia V. Bonifacci P. Marzocchi G. M. (2016). Concurrent and longitudinal predictors of calculation skills in preschoolers. *Eur. J. Psychol. Educ.* 31 155–174. 10.1007/s10212-015-0260-y

[B109] Tobia V. Bonifacci P. Marzocchi G. M. (2018). *SNUP - Senso del Numero: Prerequisiti.* Firenze: Hogrefe editore.

[B110] Torppa M. Poikkeus A. M. Laakso M. L. Eklund K. Lyytinen H. (2006). Predicting delayed letter name knowledge development and its relation to grade 1 reading achievement among children with and without familial risk for dyslexia. *Dev. Psychol.* 4 1128–1142. 10.1037/0012-1649.42.6.1128 17087547

[B111] Trochim W. M. Donnelly J. P. (2006). *The Research Methods Knowledge Base*, 3rd Edn. Cincinnati, OH: Atomic Dog.

[B112] van Steensel R. (2006). Relations between socio-cultural factors, the home literacy environment and children’s literacy development in the first years of primary education. *J. Res. Read.* 29 367–382. 10.1111/j.1467-9817.2006.00301.x

[B113] von Aster M. G. Shalev R. S. (2007). Number development and developmental dyscalculia. *Dev. Med. Child Neurol.* 49 868–873. 10.1111/j.1469-8749.2007.00868.x 17979867

[B114] Wagner J. B. Johnson S. C. (2011). An association between understanding cardinality and analog magnitude representations in preschoolers. *Cognition* 119 10–22. 10.1016/j.cognition.2010.11.014 21288508

[B115] Westermann G. Mareschal D. Johnson M. H. Sirois S. Spratling M. W. Thomas M. S. (2007). Neuroconstructivism. *Dev. Sci.* 10 75–83. 17181703 10.1111/j.1467-7687.2007.00567.x

[B116] Wilcox R. R. (2010). *Fundamentals of Modern Statistical Methods: Substantially Improving Power and Accuracy*, 2nd Edn. New York: Springer.

[B117] Wollman-Bonilla J. E. (2001). Family involvement in early writing instruction. *J. Early Child. Literacy* 1 167–192. 10.1177/14687984010012003

[B118] Xu F. Spelke E. S. Goddard S. (2005). Number sense in human infants. *Dev. Sci.* 8 88–101. 10.1111/j.1467-7687.2005.00395.x 15647069

[B119] Yuan K.-H. Chan W. Marcoulides G. A. Bentler P. M. (2016). Assessing structural equation models by equivalence testing with adjusted fit indexes. *Struc. Equat. Model.* 23 3 19–330.

[B120] Zadeh Z. Y. Farnia F. Ungerleider C. (2010). How home enrichment mediates the relationship between maternal education and children’s achievement in reading and math. *Early Educ. Dev.* 21 568–594. 10.1080/10409280903118424

